# Iatrogenic Cushing’s Syndrome from a Joint Health Supplement: Identification of Undeclared Pharmaceuticals by HRMS

**DOI:** 10.21627/2xkas668

**Published:** 2026-08-21

**Authors:** Hannah J. Lusk, Sarah Kim, Kara L. Lynch

**Affiliations:** Department of Laboratory Medicine, University of California San Francisco, San Francisco, CA, USA; Division of Endocrinology and Metabolism, Department of Medicine, University of California San Francisco, CA, USA; Department of Laboratory Medicine, University of California San Francisco, San Francisco, CA, USA

**Keywords:** Cushing’s syndrome, iatrogenic glucocorticoid exposure, dietary supplement adulteration, dexamethasone, untargeted high-resolution mass spectrometry, clinical toxicology, adrenal insufficiency, undeclared pharmaceuticals

## 1. Questions to Consider

1. What are the clinical signs that may suggest exogenous glucocorticoid exposure in patients not prescribed corticosteroids?
2. What are the limitations of routine clinical laboratory testing in detecting supplement adulterants?
3. What clinical strategies can be used to evaluate suspected exogenous glucocorticoid exposure in settings where advanced analytical testing is not readily available?

## 2. Case Presentation

A 32-year-old male with limited prior engagement in routine healthcare was newly diagnosed with diabetes and hypertension. Physical examination revealed a blood pressure of 140/85 mmHg, a body mass index (BMI) of 30 kg/m^2^, a dorsocervical fat pad, a rounded face, and central obesity. Approximately 2 years prior to presentation, he developed progressive bilateral hand pain, numbness, and weakness that were exacerbated by manual labor. Approximately 1 year prior to presentation, he initiated a non-prescription supplement labeled “Glucosamine Chondroitin” manufactured by DINA Distribuidora Naturista, after which he reported marked improvement in his musculoskeletal symptoms. During the subsequent year of supplement use, he developed progressive weight gain of approximately 40 pounds and other Cushingoid features. He had been taking two tablets of the supplement twice daily for the past year. He denied using any other medications or supplements.

His family history was significant for prediabetes and bilateral carpal tunnel syndrome in his mother and for type 2 diabetes in his maternal grandparents and uncle. Laboratory evaluation showed a non-fasting serum glucose of 237 mg/dL and a hemoglobin A1c of 11.3% (Table 1). Kidney function, electrolytes, and complete blood count were within normal limits. Given his clinical features, hypercortisolism was suspected; however, a random cortisol level was undetectable (Table 1). He was subsequently evaluated by endocrinology, and a cosyntropin stimulation test demonstrated secondary adrenal insufficiency, with an initial cortisol measurement of <0.5 μg/dL and a post-cosyntropin stimulated cortisol measurement of 4.6 μg/dL, which is less than the 18 μg/dL cutoff.

**Table 1.** Laboratory studies of this patient.

General chemistry	Serum levels	Reference intervals
Sodium (mmol/L)	136	136–145
Potassium (mmol/L)	4.5	3.5–5.1
Chloride (mmol/L)	99	98–107
CO_2_ (mmol/L)	29	20–31
BUN (mg/dL)	18	9–23
Creatinine (mg/dL)	0.39	0.6–1.1
Anion gap (mmol/L)	8	4–12
eGFRcr (mL/min/1.73 m^2^)	>120	>59
Calcium (mg/dL)	10.1	8.3–10.6
Albumin (g/dL)	5	3.4–5
Total protein (g/dL)	7.4	6.4–8.3
**Lipid profile**		
Cholesterol (mg/dL)	231	< 200
Triglycerides (mg/dL)	243	<150
HDL (mg/dL)	52	>40
LDL calculated (mg/dL)	130	<130
Non-HDL calculated (mg/dL)	179	<130
Non-Fasting glucose (mg/dL)	237	70–199
**Adrenal**		
Random cortisol (µg/dL)	<0.5	3.4–22.5
ACTH (pg/mL)	2.1	7.2–63.3
**Diabetes**		
Hemoglobin A1C (%)	11.3	<5.7
**Miscellaneous chemistry**		
Dexamethasone (ng/dL)	146.8	<50
**Comprehensive drug test**	**Compound**	**Class**
	Dexamethasone	Glucocorticoid
	Meloxicam	NSAID*
	Methocarbamol	Muscle relaxer

* NSAID: nonsteroidal anti-inflammatory drug.

## 3. Case Discussion

### 3.1. Cushing’s Syndrome Results from Chronic Glucocorticoid Excess

Cushing’s syndrome results from prolonged exposure to elevated cortisol levels and is associated with a range of physical and metabolic abnormalities. Patients commonly develop hyperglycemia, hypertension, and progressive weight gain, along with changes in body fat distribution affecting the face, neck, and abdomen. Skin findings such as facial redness, easy bruising, and violaceous striae are also frequently observed and may provide important clinical clues. Although chronic administration of exogenous glucocorticoids remains the most frequent cause, endogenous hypercortisolism may result from pituitary, adrenal, or ectopic sources. In cases of endogenous disease, adrenocorticotropic hormone (ACTH) overproduction due to a benign pituitary adenoma—referred to as Cushing’s disease—accounts for most presentations [1].

Regardless of the source, prolonged glucocorticoid exposure produces similar downstream metabolic and endocrine effects. Exogenous glucocorticoids such as dexamethasone produce similar clinical manifestations through chronic activation of glucocorticoid receptors. Dexamethasone is a potent long-acting synthetic glucocorticoid that suppresses hypothalamic-pituitary-adrenal (HPA) axis signaling through negative feedback inhibition of corticotropin-releasing hormone and ACTH secretion. Prolonged exposure may therefore result in suppressed ACTH and decreased endogenous cortisol production, potentially leading to adrenal insufficiency following withdrawal. Adrenal insufficiency refers to inadequate endogenous cortisol production, which can cause fatigue, weakness, hypotension, and potentially life-threatening adrenal crisis during physiologic stress [1].

Clinical evaluation of suspected Cushing’s syndrome begins with careful assessment of medication exposure to exclude iatrogenic causes. This assessment includes inquiring about the use of dietary supplements and herbal products. Additionally, confirmation of cortisol excess is pursued using established screening approaches, including measurement of 24-h urinary free cortisol, late-night salivary cortisol testing, or assessment of HPA axis suppression following low-dose dexamethasone administration (dexamethasone suppression test). Despite the availability of these assays, diagnosis is frequently delayed, reflecting the non-specific nature of early symptoms and the complexity of interpreting cortisol measurements in the setting of comorbid disease [1].

### 3.2. Serum Dexamethasone Level Confirmed Exogenous Source of Glucocorticoids

The temporal association between his symptom onset and initiation of an over-the-counter supplement labeled “Glucosamine Chondroitin” raised suspicion for product adulteration. Serum dexamethasone was measured by a reference laboratory (ARUP Laboratories) using quantitative liquid chromatography-tandem mass spectrometry (LC-MS/MS). The measured concentration was 146.8 ng/dL (reference interval for individuals not receiving dexamethasone, <50 ng/dL), confirming exogenous dexamethasone exposure (Table 1)*.* The patient’s suppressed ACTH concentration was consistent with dexamethasone-induced suppression of the HPA axis. Subsequent analysis of the supplement was performed using liquid chromatography–high-resolution quadrupole time-of-flight mass spectrometry (LC-HRMS) with untargeted full-scan data acquisition and targeted screening against an expanded in-house mass-spectral library containing >5000 exogenous small molecules [2]. Supplement tablets were crushed and extracted in methanol (0.1 mg/mL) prior to LC-HRMS analysis. The testing revealed dexamethasone, meloxicam, a nonsteroidal anti-inflammatory drug (NSAID), and methocarbamol, a muscle relaxant, none of which were listed on the product label. A remnant sample from the patient was no longer available for testing at the time of supplement analysis. The patient was instructed to discontinue the supplement immediately and was started on a tapering course of hydrocortisone. Because adrenal insufficiency may occur following chronic exogenous glucocorticoid exposure, repeat assessment of the HPA axis, including cosyntropin stimulation testing to evaluate adrenal reserve and recovery of cortisol responsiveness, was scheduled for 6–8 weeks. Failure of serum cortisol to appropriately increase following cosyntropin stimulation is consistent with persistent adrenal insufficiency due to chronic ACTH suppression [1].

### 3.3. Dexamethasone-Adulterated Supplements Are Well Documented in the Literature

Iatrogenic Cushing’s syndrome resulting from steroid-adulterated supplements has been repeatedly documented in the literature. Large clinical series and pharmacovigilance studies have reported dozens of affected patients, with dexamethasone identified as the most frequently detected glucocorticoid adulterant in non-prescription products, particularly those marketed for pain and joint disease [3,4]. Multi-patient case series with biochemical confirmation of dexamethasone exposure have been reported in U.S. clinical settings, indicating that this remains an ongoing and clinically relevant problem rather than an isolated phenomenon [5]. At the individual level, published case reports illustrate the heterogeneous and often prolonged clinical course associated with unrecognized exposure to adulterated products. One such case described a 42-year-old man who presented with fatigue, hyperphagia, weight gain, polyuria, skin infections, and proximal muscle weakness. Examination revealed classic Cushingoid features, and laboratory testing demonstrated suppressed cortisol levels. His symptoms persisted for more than a year while he continued to take “traditional medicines” purchased over-the-counter; following counseling regarding the adulterated contents of these products, he was initiated on a corticosteroid taper to support recovery [6].

### 3.4. Artri King, an Arthritis Supplement, Has Also Been Implicated in Glucocorticoid Toxicity

In 2022, the U.S. Food and Drug Administration issued a warning regarding the supplement “Artri King,” marketed for arthritis and widely available through major retailers, including Amazon and Walmart. Analysis revealed multiple undeclared pharmacologic agents, including dexamethasone, diclofenac, and methocarbamol [7]. A subsequent case series linked Artri King use to HPA axis disruption in three individuals [5]. One patient presented with hyponatremia, weight gain, muscle weakness, a dorsocervical fat pad, and red striae; another exhibited overt Cushingoid features with low cortisol levels; and a third had poorly controlled diabetes accompanied by elevated serum dexamethasone. Two of the three patients required glucocorticoid tapering to mitigate the risk of adrenal crisis, whereas the third discontinued the supplement without tapering because of brief prior exposure. Additional evidence of endocrine disruption was reported in a study of 13 patients presenting to an orthopedic surgery clinic while taking Artri King. One patient developed new-onset diabetes, and nine were found to have low serum cortisol levels, including two with concomitantly low ACTH. These findings prompted endocrine referral and recommendations for perioperative stress-dose steroid coverage [8].

### 3.5. Corticosteroid Adulteration in Pain-Relief Supplements Poses Substantial Clinical Risk

A recent review identified corticosteroids and NSAIDs as the most common adulterants in supplements marketed for pain relief, both of which were detected in this case [9]. Dexamethasone, in particular, has been highlighted as a frequently encountered contaminant [9]. In a screen of 116 herbal products, dexamethasone was identified in 16 samples, with no other adulterants identified [10]. These substances are likely added to enhance perceived efficacy by suppressing inflammatory symptoms. However, chronic exposure to corticosteroids can result in adrenal atrophy, and potentially life-threatening adrenal crisis if the corticosteroid is abruptly withdrawn, underscoring the need for gradual tapering and close clinical monitoring [11,12]. Additionally, exposure to undisclosed NSAIDs may increase the risk of cumulative NSAID toxicity, particularly in patients simultaneously using prescribed or over-the-counter NSAID-containing medications. Potential complications include gastrointestinal bleeding, renal injury, and cardiovascular toxicity [13].

### 3.6. Detection of Supplement Adulterants Is Challenging without HRMS

Despite the clinical importance of identifying adulterants, most clinical laboratories are not equipped to perform comprehensive drug testing in biological samples or dietary supplements. This limits the timely identification of hidden ingredients and delays diagnosis in patients with atypical presentations. Drug testing using HRMS offers a valuable solution for detecting unexpected compounds in complex cases, particularly in cases where exposure to adulterated supplements, novel psychoactive substances, or off-label medications is suspected. Unlike targeted panels or immunoassays, HRMS provides broad-spectrum compound detection, retrospective data mining, and high mass accuracy for structural identification. In this case, rapid identification was facilitated through targeted screening against a large in-house spectral library following untargeted full-scan HRMS data acquisition. In cases where compounds are not represented in existing libraries, additional untargeted data interpretation may be required, including review of accurate mass measurements, fragmentation spectra, and comparison with external spectral databases. Definitive confirmation may require acquisition and analysis of analytical reference standards, which increases turnaround time before clinical reporting. As demonstrated in this case, HRMS can confirm the presence of non-disclosed pharmaceuticals and guide clinical management. However, implementation in routine clinical laboratories is limited because of cost, specialized training requirements, and a lack of standardized workflows [14]. Broader adoption of HRMS-based untargeted drug testing in clinical laboratories could significantly enhance the diagnostic evaluation of unusual clinical presentations and improve patient safety in the face of a growing and underregulated supplement market.

### 3.7. Limited Access to HRMS Necessitates Heightened Clinical Suspicion

Because untargeted HRMS is not available at most institutions, clinicians should maintain a high index of suspicion for exogenous glucocorticoid exposure in patients with overt Cushingoid features or adrenal suppression who are not prescribed corticosteroids. This case underscores the importance of obtaining a thorough clinical history, including a detailed inquiry into medication and supplement use, particularly non-prescription and herbal products. When adulteration is suspected, measurement of serum levels for common glucocorticoids through reference laboratories may provide confirmatory evidence. Although discontinuation of the suspected supplement may be considered, this must be approached with caution because abrupt withdrawal in the setting of adrenal insufficiency can precipitate a potentially life-threatening adrenal crisis. Until the source of exposure is clarified, cautious management with close endocrinology follow-up is warranted.

## 4. Case Resolution

Following identification of exogenous glucocorticoid exposure, the patient was initiated on physiologic hydrocortisone replacement with plans for a gradual taper. Because of persistent musculoskeletal pain and symptoms consistent with glucocorticoid withdrawal, dose reductions were repeatedly unsuccessful, and a slower taper over several months was required given the approximately 1-year duration of exposure. As of the most recent follow-up, the patient remains on hydrocortisone replacement and tapering efforts are ongoing.

## 5. Educational Points / Key Learning Points / Points of Interest

- Herbal supplements may include undisclosed pharmaceuticals, including glucocorticoids.
- Iatrogenic Cushing’s syndrome can result from chronic exposure to glucocorticoid-adulterated supplements.
- Sudden withdrawal of corticosteroids can lead to adrenal insufficiency and adrenal crisis, requiring careful tapering.
- Routine toxicology screens may miss adulterants that are not part of standard panels.
- Untargeted drug testing with HRMS enables detection of unexpected compounds and supports accurate clinical diagnosis.

## 6. Conclusion

This case highlights the diagnostic value of untargeted HRMS in identifying pharmaceutical adulterants in biological samples or dietary supplements and underscores the need for expanded access to such testing in clinical laboratories.

## Data Availability

Raw LC–MS/MS data supporting the findings of this study, including HRMS data, have been deposited in the MassIVE repository (University of California, San Diego). The dataset is available under accession number MSV000102496 and can be accessed at ftp://massive-ftp.ucsd.edu/v13/MSV000102496/.

## Abbreviations

ACTH: adrenocorticotropic hormone
BMI: body mass index
HPA: hypothalamic-pituitary-adrenal
LC-HRMS: liquid chromatography–high-resolution quadrupole time-of-flight mass spectrometry
LC-MS/MS: liquid chromatography-tandem mass spectrometry
NSAID: nonsteroidal anti-inflammatory drug

## References

[1] Reincke M, Fleseriu M. Cushing syndrome: A review. JAMA 2023;330:170-81. 10.1001/jama.2023.11305 37432427

[2] Colby JM, Lynch KL. Drug screening using liquid chromatography quadrupole time-of-flight (LC-QqTOF) mass spectrometry. Methods Mol Biol. 2019;1872:181-90. 10.1007/978-1-4939-8823-5_17 30350290

[3] Xu Y, Patel DN, Ng SLP, Tan SH, Toh D, Poh J, et al. Retrospective study of reported adverse events due to complementary health products in Singapore from 2010 to 2016. Front Med (Lausanne). 2018;5:167. 10.3389/fmed.2018.00167 PMC6006675 29946545

[4] Chong YK, Ching CK, Ng SW, Mak TWL. Corticosteroid adulteration in proprietary Chinese medicines: A recurring problem. Hong Kong Med J. 2015;21:411-6. 10.12809/hkmj154542 26314568

[5] Berg EA, Dao L, Yu R, Hurtado C. Artri King-induced hypothalamic-pituitary-adrenal axis disruption: A report of 3 cases. JCEM Case Rep. 2023;2:luad154. 10.1210/jcemcr/luad154 PMC10729854 38116163

[6] Asamoah KT, Akpalu J, Atiase Y, Yorke E. A case of Cushing syndrome due to accidental intake of dexamethasone: A call for enforcement of regulatory laws. Ghana Med J. 2021;55:152-5. 10.4314/GMJ.V55I2.9 PMC12410028 40917378

[7] US Food and Drug Administration. Public notification: Artri King contains hidden drug ingredients. 2022. Available from: https://www.fda.gov/drugs/medication-health-fraud/public-notification-artri-king-contains-hidden-drug-ingredients

[8] Culler M, Mayfield CK, Aron A, Del Val L, Longjohn D, Heckmann ND. Endocrinologic abnormalities observed among total joint arthroplasty patients using “Artri King” and related over-the-counter supplements: A cautionary tale from a safety net hospital. J Clin Med. 2024;13:7240. 10.3390/jcm13237240 PMC11641894 39685700

[9] Qurrotul Ainiyah E, Anggia V. Review of adulterated herbal products and supplements and methods of analysis. Pharmaceutical and Biomedical Sciences Journal. 2020;2:53-64. 10.15408/pbsj.v2i2.18328

[10] Kim NS, Kim J, Lim NY, Lee JH, Park S, Kang H. Simultaneous determination of illegal drug substances in dietary supplements for gout and osteoporosis using ultra-performance liquid chromatography and liquid chromatography-quadrupole-time-of-flight mass spectrometry. J Pharm Biomed Anal. 2020;179:113003. 10.1016/j.jpba.2019.113003 31816474

[11] Gireesh KM, Sushma Priya MS, Sanjeev M, Prajwal, Manjunatha R, Vinod N. Case report on dexamethasone induced iatrogenic Cushing syndrome. Ann Clin Case Rep. 2023;8:2468.

[12] Pelewicz K, Miśkiewicz P. Glucocorticoid withdrawal—an overview on when and how to diagnose adrenal insufficiency in clinical practice. Diagnostics. 2021;11:728. 10.3390/DIAGNOSTICS11040728 PMC8072923 33923971

[13] Harirforoosh S, Asghar W, Jamali F. Adverse effects of nonsteroidal antiinflammatory drugs: An update of gastrointestinal, cardiovascular and renal complications. J Pharm Pharm Sci. 2013;16(5):821-47. 10.18433/J3VW2F 24393558

[14] Maurer HH. Pitfalls in drug testing by hyphenated low- and high-resolution mass spectrometry. Drug Test Anal. 2020;12:172-9. 10.1002/dta.2744 31804756

